# Evaluation of primary adnexal masses by 3T MRI: categorization with conventional MR imaging and diffusion-weighted imaging

**DOI:** 10.1186/1757-2215-5-33

**Published:** 2012-11-14

**Authors:** He Zhang, Guo-Fu Zhang, Zhi-Yan He, Zheng-Yu Li, Ming Zhu, Gui-Xiang Zhang

**Affiliations:** 1 Department of Radiology, Shanghai First People’s Hospital, Medical College, Shanghai Jiaotong University, No. 100, Hai Ning Road, Shanghai, 200080, China; 2 Department of Radiology,Obstetrics and Gynecology Hospital, The Affiliated hospital of Fudan University, No. 419, Fang xie Road, Shanghai, 200011, China

**Keywords:** Magnetic resonance images, Diffusion weighted imaging, 3.0 Tesla, Ovary diseases

## Abstract

**Background:**

To investigate the 3.0-Tesla (3 T) magnetic resonance imaging (MRI) characteristics of primary adnexal lesions for discriminating benign from malignant lesions.

**Methods:**

One hundred thirty-nine patients with pathologically proven primary adnexal masses referred for 3 T MRI assessment preoperatively were included. Baseline characteristics, components, and conventional MRI and diffusion-weighted imaging (DWI-MRI) signals were recorded and compared.

**Results:**

There were 22 ovarian cysts, 33 endometriomas, 43 benign tumors and 42 malignant tumors. When ovarian cyst and endometrioma were excluded, there were no significant differences in patients’ age between benign and malignant tumor (*P* = 0.235). There were no significant differences (*P* = 0.606) in the conventional MRI signals and significant difference (*P* = 0.008) in DWI-MRI signal between the non-malignant and malignant lesions. There was a significant difference (*P* = 0.000) in the apparent diffusion coefficient values (ADCs) between the non-malignant and malignant lesions.

**Conclusions:**

3 T MRI categorized the characteristics of primary adnexal lesions. Conventional MRI signals were not useful for characterizing between benign and malignant lesions. DWI-MRI and ADCs were helpful for distinguishing malignant from benign ovarian lesions.

## Background

The prevalence of adnexal masses in the general population is 0.17%–5.9% in asymptomatic women and 7.1%–12% in symptomatic women in the United States [1]. Correct determination of the aetiology of adnexal lesions before surgery may help in the choice of optimal treatment and improve patient care by avoiding aggressive therapy. Sonography is routinely used clinically because of its low expense and easy and rapid maneuverability. Nevertheless, its limited capability of characterizing tissues makes it an unsuitable modality for complex adnexal masses and staging of ovarian cancers [2]. With the advantages of superb tissue contrast resolution and no ionizing radiation, magnetic resonance imaging (MRI) is generally performed for problem solving in the assessment of indeterminate sonographic adnexal masses [3-5] with a sensitivity of 92% and specificity of 85% for the detection of malignant adnexal tumors [1,5]. However, because of the widely overlapping imaging characteristics of benign and malignant ovarian tumors, even experienced gynaecological radiologists may have difficulty in making a confident diagnosis before surgery [6]. With advancements in MRI technology, diffusion-weighted imaging (DWI-MRI) applied in gynaecological diseases also has been reported in several studies with promising results [7-9]. Compared with 1.5 T, the signal-to-noise ratio at 3 T has been increased and background suppression has been improved, which allows better categorization of variable components of ovarian diseases [10,11]. Studies with focus on primary adnexal lesions evaluated by 3.0 T (3 T) still are limited to date. The main purpose of this study was to describe the conventional MRI and DWI-MRI characteristics of primary adnexal lesions at 3 T and compare with those of 1.5 T MR previously reported.

## Methods

### Study subjects

From Jan 2010 to Jun 2012, 312 consecutive patients with clinically suspected adnexal diseases underwent 3.0 T MRI examinations before pelvic or laparoscopic surgery. The time interval between MRI evaluation and surgery was within 1 month. Patients with metastatic ovarian tumors (n = 32), recurrent diseases of known gynaecological malignancies (n = 55), previous treatment history (n = 30) and a lack of DWI-MRI (n = 3) were excluded. Patients with mature teratoma (n = 53) were also arbitrarily excluded because diagnosis is less challenging, as described in the literature [9]. Therefore, we retrospectively reviewed 139 patients (13–83 years of age; average age, 51.3 ± 17.1 years) with pathologically proven primary adnexal masses. The patient cohort group included 22 ovarian cysts, 33 endometriomas and 43 benign and 42 malignant tumors. Details of the patients are summarized in Table 1. Our institutional review board approved this study, and waivers of informed consent from all participants were granted.

**Table 1:** The details of 140 primary adnexal lesions proven on histopathological diagnosis in 139 patients

**Histolopathological diagnosis**	**Numbers**	**ADC Values (10**^**-3**^**/mm**^**2**^**/s)**^**▲**^
Ovarian cyst	22	2.47 ± 0.93
**Endometrioma**	**33**	1.75 ± 0.71
**Benign**	**43**	2.03 ± 0.94
Brenner tumor	3	0.80 ± 0.22
Struma ovarii	4	2.58 ± 0.31
Fibroma	6	1.58 ± 1.09
Theca cell tumor	7	1.46 ± 0.50
Serous cystadenoma	9	2.51 ± 0.66
Mucinous cystadenoma	14	2.30 ± 1.02
**Malignant**	**42**	1.39 ± 0.62
Granular cell tumor	1	0.68
Embryonal carcinoma	1	1.31
Clear cell adenocarcinoma	5	1.25 ± 0.37
Endometrioid adenocarcinoma	5	1.06 ± 0.60
Leiomyosarcoma	1	0.86
Undefined adenocarcinoma	2	1.14 ± 0.41
Dysgerminoma	1	1.16
Mixed germ cell tumor	1	2.63
Serous cystadenocarcinoma	12	1.12 ± 0.42
Mucinous cystadenocarcinoma	4	2.11 ± 0.30
Borderline serous or mucinous cystadenoma	9	1.90 ± 0.58

^▲^ indicates mean values ± standard deviation.

### Image acquisition

All MRI examinations were performed on a 3.0-Tesla (3 T) system (Signa HD, General Medical Systems, GE, USA) equipped with an 8-channel cardiac array coil. The scan range was from the umbilicus level to the pubic symphysis in the caudocranial direction. For any larger lesion that could not be covered on axial imaging, a sagittal scanning sequence was performed to include as much of the entire lesion as possible. Routine MRI protocols were used for the assessment of adnexal masses, which included the following: axial fast spin-echo (FSE) T1-weighted images (T1WI), sagittal FSE T2WI and fat-suppressed T2WI (FS T2WI). A DWI-MRI sequence used an echo-planar imaging sequence with an array spatial sensitivity encoding technique. The parameter details of the T1WI MR protocol were: repetition time (TR), 460 ms; echo time (TE), 10 ms; NEX, 2; and thickness, 6.0 mm. The parameter details of the T2WI MR protocol were: TR, 2400 ms; TE, 85 ms; NEX, 1; and thickness, 6.0 mm. The parameter details of the FS T2WI MR protocol were: TR, 3160 ms; TE, 90 ms; NEX, 2; and thickness, 6.0 mm. The parameter details of the DWI-MRI MR protocol were: TR, 3500 ms; TE, 61 ms; NEX, 6; and thickness, 6.0 mm. and, the *b* value = 0 and 700 s/mm^2^. A liver acquisition with volume acceleration (LAVA) sequence was used to perform contrast-enhanced pelvic imaging, and a power injector (Missouri Ulrich; Ulm, Germany) was used for injection of contrast material (Magnevist, Bayer Schering Pharma AG, Germany). The parameter details of the LAVA MR protocol were: TR, 3.4 ms; TE, 1.4 ms; NEX, 1; flip angle, 1.5; and band width, 125 kHz. Images were acquired at multiple phases of contrast medium enhancement in both sagittal and axial planes (precontrast sagittal and axial oblique and postcontrast at 20 seconds, 40 seconds, 60 seconds and 80 seconds in the axial plane and 120 seconds in the sagittal plane).

### MRI image analysis

The MRI characteristics of each adnexal lesion were separately recorded according to the following items: 1) lesion components graded on a 5-point scale [1 = cystic; 2 = solid; 3 = cyst with septum; 4 = cyst with solid components (nodules); 5 = cyst with septum and solid components; 2) signal intensity compared with that of the outer myometrium on T1WI and T2WI graded on a 9-point scale (low or equal intensity on T1WI and high intensity = 1, low intensity = 2 and mixed signal = 3 on T2WI, high or equal intensity on T1WI and high intensity = 4, low intensity = 5 and mixed signal = 6 on T2WI, mixed signal on T1WI and low intensity = 7, high intensity = 8, and mixed signal intensity = 9 on T2WI) and 3) DWI-MRI signal graded on a 4-point scale (1 = low; 2 = intermediate; 3 = high; 4 = mixed). The low signals on DWI-MRI indicated that the signals of lesion were similar with the pelvic bone signal and the intermediate signals were similar with the outer myometrium. The high signals on DWI-MRI were like that of endometrium.

Commercially available software was used on a postprocessing workstation (GE Advantage workstation 4.3, General Electric Healthcare, Milwaukee, WI, USA) to calculate ADCs. Regions of interest (ROI) were drawn manually in both cystic and solid areas with no more than 3 sites in each lesion on *b* value = 700 DWI-MRI images. The round or elliptical circle were centrally placed in the targeted region with the area range of 160–220 mm^2^. Only the lowest ADCs were used for subsequent statistical analysis. For any lesions which were not clearly depicted on DWI-MRI, then axial fat-suppressed T2WI images and dynamic contrast-enhanced images were also reviewed in order to help to define the small nodular components. ADCs were not available for small size (< 10 mm) in endometric cyst group (one case) or severe pulsative artefacts due to large size in benign tumors (three cases ≥ 100 mm) and malignant tumors (four cases ≥ 250 mm).

Two observers (Y. Z. H. and H.Z, with 15 and 6 years of experience in gynaecological imaging, respectively), who were blinded to the histological results independently analysed all of the MRI datasets of each participant on a Picture Archiving and Communication System terminal server. In patients with ≥ 1 lesions with different pathological type, all lesions were selected for further analysis. For interobserver discrepancies in the evaluation of adnexal lesions, consensus was achieved or a majority decision was obtained.

### Statistical analyses

Continuous variables were expressed as the means ± standard deviation (SD) and compared with the unpaired *t*-test if normally distributed or the Mann–Whitney test if not normally distributed. A nonparametric test (Mann–Whitney) was used to test the components and signals of the lesions and the DWI-MRI signal within each group. SPSS (version 13.0, SPSS Inc., Chicago, USA) was used to perform statistical analyses.

## Results and discussion

In these 140 samples, the mean patient age in the endometrioma group was lower than that in the other groups. The patients’ mean age and maximum diameter of the lesions in the endometrioma group differed significantly with the benign and malignant ovarian tumors. In the ovarian cyst group, the mean age was significantly different from that of the endometrioma group (*P* = 0.000) but did not differ from that of the other groups. The maximum diameter of the ovarian cyst was similar to that of the endometrioma (*P* = 0.172), smaller than that of the benign lesions (*P* = 0.032) and much smaller than that of the malignant lesions (*P* = 0.000). When ovarian cyst and endometrioma were excluded, there were no significant differences in age between the benign and malignant tumors (*P* = 0.235). The CA125 level in the ovarian cyst group was obviously less than that in the other groups. The CA125 level in the endometrioma group was higher than that in the benign tumor group (*P* = 0.000) and less than that in the malignant group, but the difference was not significant (*P* = 0.400). When endometriomas were excluded, there was still a significant difference in the CA125 level between the benign and malignant tumors (*P* = 0.000). The basic characteristics of the studied patients are summarized in Table 2 and Table 3.

**Table 2:** Baseline characteristics and ADC values of 139 patients with pathologically proven primary adnexal masses

**Histology diagnosis**	**Numbers**	**Age (year)**	**Maximum Diameters (mm)**	CA125*	**ADC (10**^**-3**^**/mm**^**2**^**/s)**^▲^
Ovarian cyst	21	52.2 ± 15.3	58.9 ± 31.2	36.3 ± 51.2	2.47 ± 0.93
Endometrioma	33	37.0 ± 10.4	49.2 ± 20.4	78.5 ± 91.6	1.75 ± 0.71
Benign tumor	43	58.8 ± 17.1	82.1 ± 54.1	42.4 ± 88.0	2.03 ± 0.94
Malignant tumor	42	54.5 ± 16.0	108.6 ± 57.2	310.4 ± 745.4	1.39 ± 0.62

* CA125 values were not unavailable in 31 cases.

^▲^ indicates mean values ± standard deviation.

**Table 3:** **The statistically significant difference (*p *value) of baseline characteristics and ADC values within four groups in 139 patients with pathologically proven primary adnexal masses**

	Age*	Maximum Diameters*	**CA125**^**▲**^**level (U/mL)**	ADC* (10^**-3**^**/mm**^**2**^**/s)**
Ovarian cyst & Endometrioma	0.000	0.211	0.001	0.003
Ovarian cyst & Benign tumor	0.119	0.032	0.509	0.080
Ovarian cyst & Malignant tumor	0.570	0.000	0.001	0.000
Endometrioma & Benign tumor	0.000	0.001	0.000	0.121
Endometrioma & Malignant tumor	0.000	0.000	0.400	0.032
Benign group & Malignant group	0.235	0.031	0.000	0.000
Non-malignant & Malignant	0.065	0.000	0.000	0.000

* Unpaired *t*-test was used to test the significant difference.

^▲^ Mann–Whitney test was used to test the significant difference.

### Conventional MRI lesions appearance

**Figure 1:**
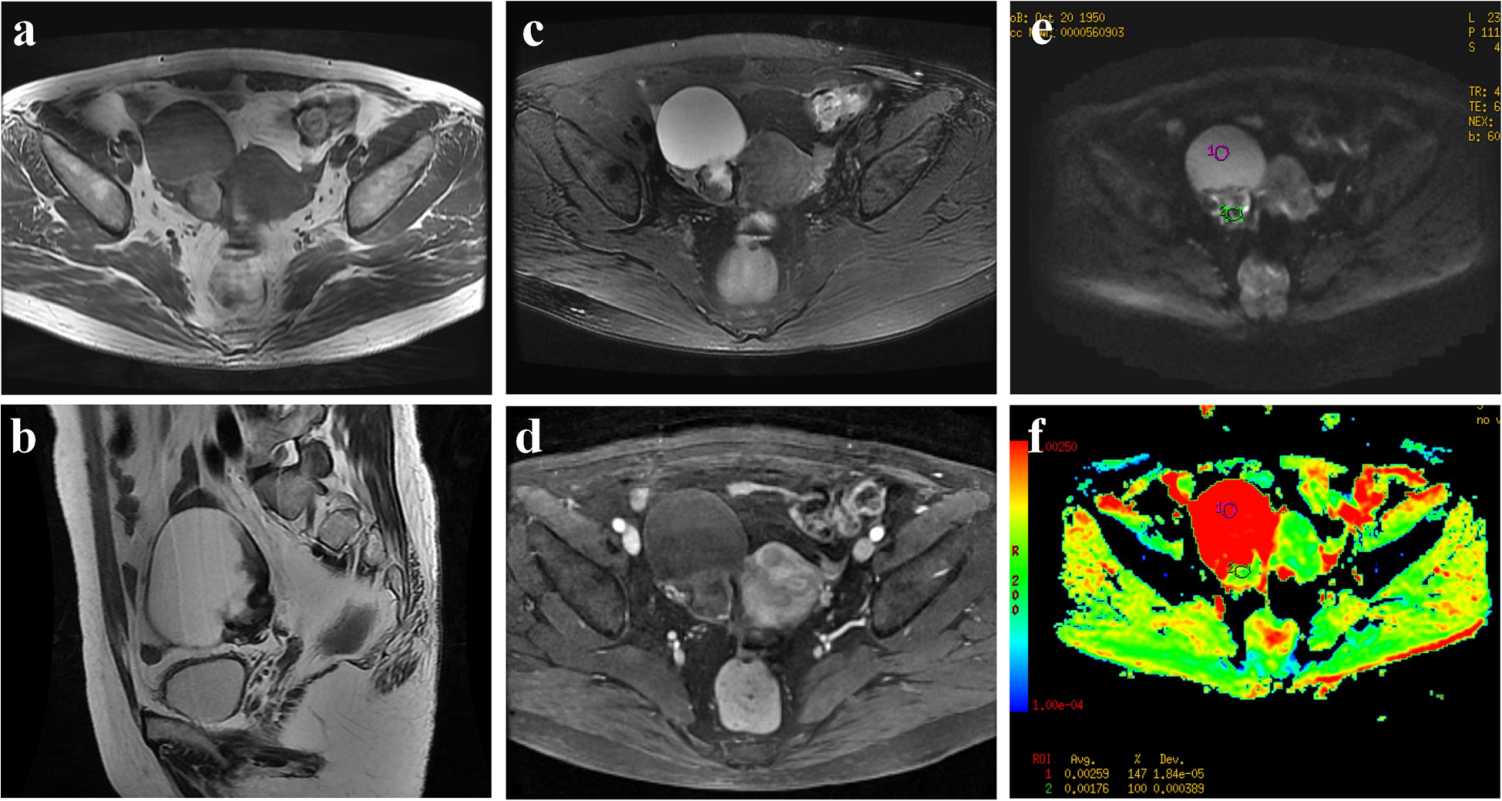
**A 60-year-old female patient with histological proven hemorrhagic ovarian cyst.** (**a**) Axial T1WI reveals an inhomogeneous cystic mass with mostly isointensity in the right adnexal region. (**b**) On sagital T2WI, the cystic component of the tumor is homogenous hyperintensity, while the debris of hemorrhagic components are iso-hypo intensity, morphologically mimicking vegetations on the wall. (**c**) On fat-suppressed T2WI, the signal of mass is similar with (**b**). (**d**) The lesion shows weakly, marginally enhancement on contrast-enhanced fat-suppressed T1WI. (**e**) On DWI-MRI (*b* = 700 s/mm^2^), the lesion upwardly appears as the homogeneous hyperintensity and downwardly as inhomogenous isointensity. (**f**) ADC map demonstrates marked hyperintensity of the cystic components (T2 shine-through effect) and isointensity of solid components. The ADC values at the corresponding site are 2.59 ×10^-3^ (cyst) and 1.76 ×10^-3^ (solid), respectively.

**Figure 2:**
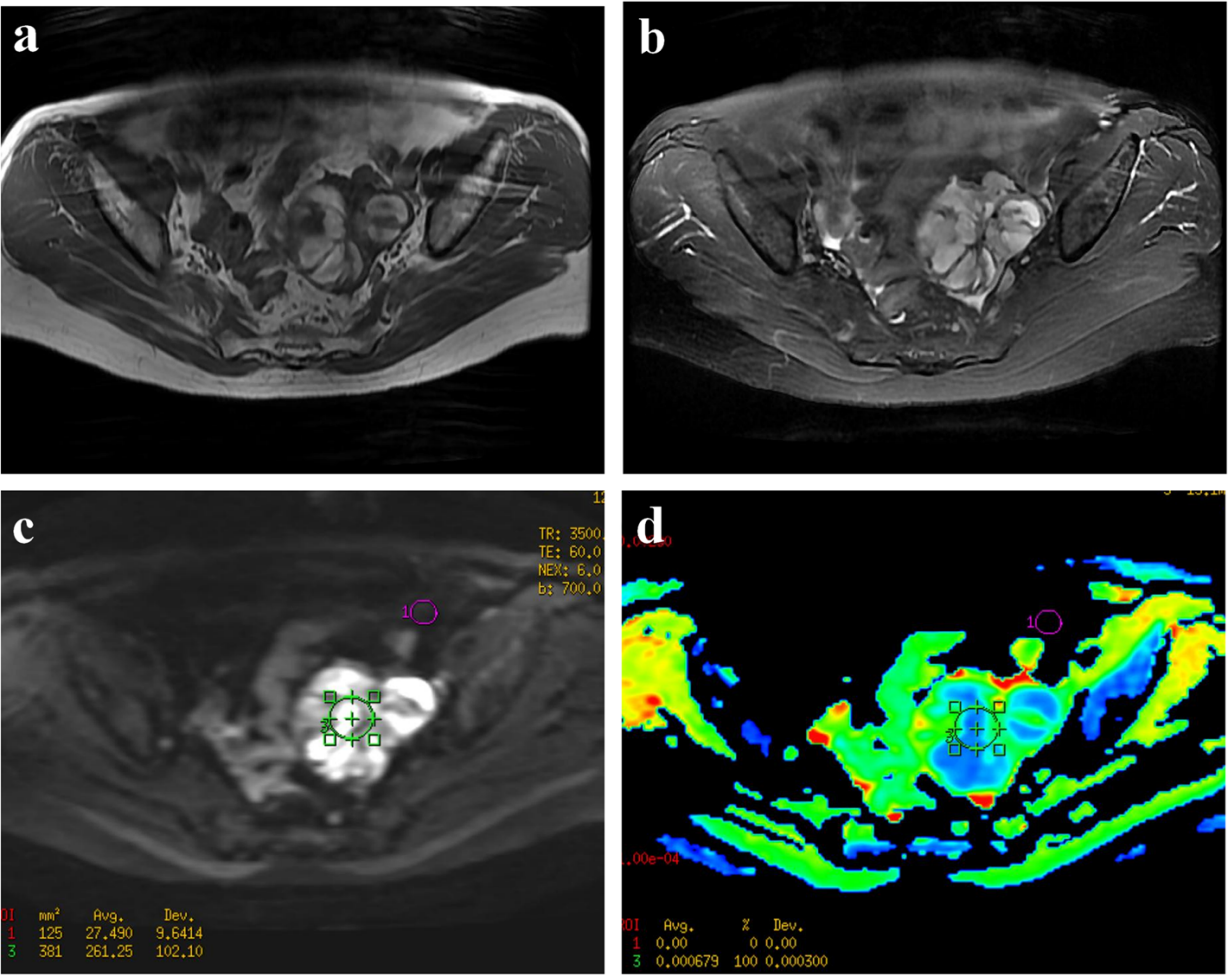
**A 60-year-old female patient with Granular cell tumor.** (**a**) Axial T1WI reveals a well-demarcated solid tumor with hyperintensity in the left adnexal region. (**b**) On fat-suppressed T2WI, the mass shows mixed hyperintensity. (**c**) On DWI-MRI (*b* = 700 s/mm^2^), the lesion appears as the inhomogeneous hyperintensity. (**d**) ADC map demonstrates marked hypointensity of the solid components. The ADC value at the corresponding site is 0.68x10^-3^.

Most of ovarian cyst (18/22) (Figure 1) and endometrioma (27/33) appeared as cystic lesions. Most of the benign tumors appeared as lesions that were solid (14/43) or cyst with septa (14/43). A cystic component combined with a solid nodule (22/42) was the most common type in the malignant group. There were 15 solid lesions in benign and 7 solid lesions in malignant group (Figure 2). There was significant difference in signal between the benign and malignant tumor (*P* = 0.000). The MRI components within the 4 groups in 140 primary adnexal lesions are summarized in Table 4.

**Table 4:** The MRI components within four groups in 140 primary adnexal lesions

**Pathology Type**	**Cystic**	**Solid**	**Cyst with septum**	**Cyst with solid**	**Cyst with septum and solid**	**Total**
Ovarian cyst	18			3	1	22
Eendometrioma	27	1	4		1	33
Benign tumor	8	14	14	2	5	43
Malignant tumor	2	7	2	22	9	42
Total	55	22	20	27	16	140

### Conventional MRI signal character

Most of ovarian cyst (13/22) showed as low/equal intensity on T1WI and mixed signal on T2WI. Most of endometrioma (17/33) appeared as high/equal intensity on T1WI and hyper intensity on T2WI. Accordingly, most of the benign (29/43) and malignant tumors (19/42) showed low/equal intensity on T1WI and hyper intensity on T2WI. The MRI signals between ovarian cyst and endometrioma and benign tumor gave statistically significant differences (*P* = 0.000, *P* = 0.000), while no difference was observed between ovarian cyst and malignant tumor (*P* = 0.965). There was a significant difference between the endometrioma and benign and malignant tumor (*P* = 0.000, *P* = 0.002). The signal difference between benign and malignant tumor also significantly differed (*P* = 0.008). The MRI signals of the lesions were not significantly different between the non-malignant (regarding the ovarian cyst, endometrioma and benign ovarian tumor as the whole category) and malignant lesions (*P* = 0.606). The MRI signal characteristics within the 4 groups are summarized in Table 5.

**Table 5:** The MRI signal characteristics within four groups in 140 primary adnexal lesions

	**T1WI Low and/Equal intensity**			**T1WI High and/Equal intensity**			**T1WI Mixed intensity**			**Total**
**T2WI Pathology Type**	**Hyper**	**Hypo**	**Mixed**	**Hyper**	**Hypo**	**Mixed**	**Hyper**	**Hypo**	**Mixed**	
Ovarian cyst	4	3	13	2						22
Endometrioma	3	2		17	7	2			2	33
Benign tumor	29	8		5					1	43
Malignant tumor	19	4		13		1			5	42
Total	55	17	13	37	7	3			8	140

### DWI-MRI character

Most of the primary adnexal lesions (69/140) showed hyperintensity on DWI-MRI. Most of the malignant tumors (27/42) gave high signal in this series. The differences in DWI-MRI signals within each group were statistically significant except between endometrioma and malignant tumor (*P* = 0.546). However, the DWI-MRI signals of the lesions were significantly different between the non-malignant and malignant groups (*P* = 0.008). The DWI-MRI signals within the 4 groups are summarized in Table 6.

**Table 6:** The DWI signals within four groups in 140 primary adnexal lesions

**Pathology Type**	**Low**	**Intermediate**	**High**	**Mixed**	**Total**
Ovarian cyst	12	3	4	3	22
Endometrioma	3		20	10	33
Benign tumor	6	14	18	5	43
Malignant tumor	1	4	27	10	42
Total	22	21	69	28	140

**Figure 3:**
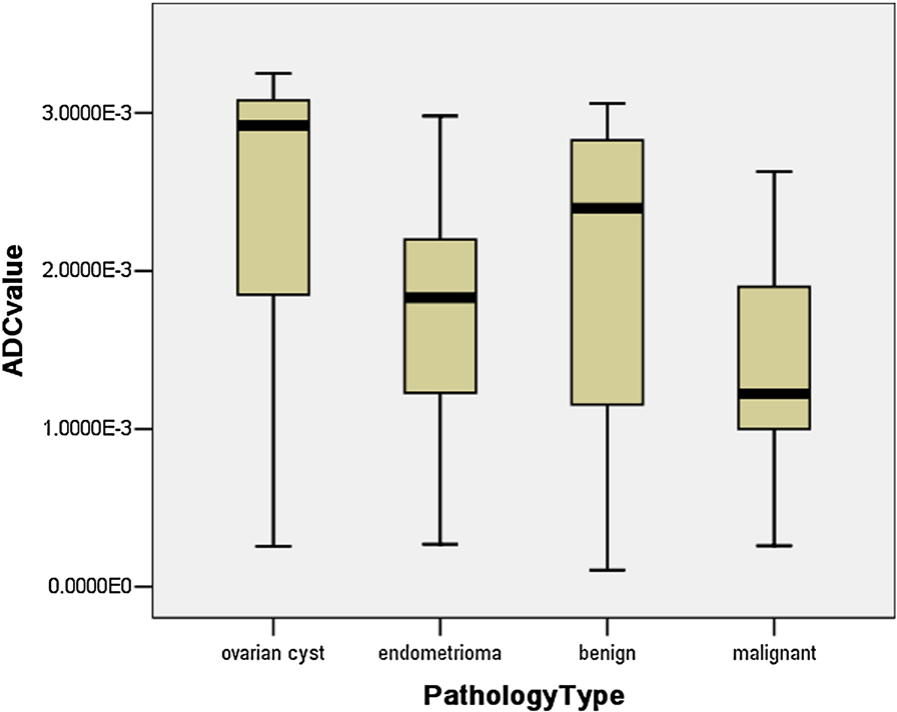
**Stem-and-Leaf Plots of the calculated ADC values (10**^**-3**^**/mm**^**2**^**/s) within four groups.** The mean ADC values in ovarian cysts were higher than that in other three groups. The mean ADC values in benign tumor were obviously higher than that in malignant tumor (*p* = 0.000), while wide overlaps with endometrioma (*p* = 0.121).

In our results, the highest ADC value was 2.47 ± 0.93 observed in ovarian cyst, while the lowest was 1.39 ± 0.62 in the malignant tumor (Table 1). The mean ADC value of ovarian cysts was higher than that of the other groups, although a significant difference between the ovarian cyst and benign tumors was not observed (*P* = 0.080). No significant differences were observed in the ADCs between the endometrioma and benign (*P* = 0.121), but a significant difference was observed between the benign and malignant tumor (*P* = 0.000) (Figure 3). There was also a significant difference between the non-malignant and malignant lesions (*P* = 0.000). In the 22 solid lesions in this sample (Figure 2), the mean ADCs between the benign and malignant tumors were not significantly different (*P* = 0.399).

Primary adnexal masses comprise a broad spectrum of diseases in which the aetiology is often difficult to determine by ultrasonography because of the similarity in the imaging appearances. With the advantage of excellent contrast resolution, MRI is a problem-solving modality for distinguishing benign from malignant ovarian tumors [12-16].

We reported our single-centre preliminary experiences using 3.0 T MRI for evaluation of primary adnexal lesions in 139 subjects. With respect to baseline characteristics of primary adnexal lesions, when ovarian cyst and endometrioma were excluded, there was significant difference in maximum diameters of lesions and CA125 level between the primary ovarian benign and malignant tumors. CA125 level could be used as a reference standard for differentiating benign from malignant tumors [17], although some overlap between endometrioma and malignant tumors was observed.

Considering the components of lesions, our findings are in line with those of other studies that used conventional imaging and showed that complex structures were more often seen in ovarian tumors and there was more overlap in appearance between benign and malignant tumors [2,18-21]. In our series, except for ovarian cyst, the signal intensity in all primary ovarian lesions varied considerably, which would present a diagnostic challenge if conventional imaging only were used. The typical shading character (high signal on T1WI and low signal on T2WI) of endometrioma, as well described in the literature [22], does not often occur in this patient group. It has been reported that endometriosis is a precursor lesion of ovarian malignancies, particularly in endometrioid and clear-cell types [2,23]. Considering this, caution is needed to precisely confirm the diagnosis of endometrioma, especially in cases with atypical imaging findings.

DWI-MRI is a functional imaging technique that is now widely applied for characterization of primary adnexal lesions and staging of ovary cancers [7,9,24,25]. However, DWI-MRI in gynaecological adnexial masses must be carefully interpreted by diagnosticians because any normal structures could appear bright on high *b*-value images [26,27]. Two studies reported that the areas of high signal intensity on DWI-MRI were observed more often in malignant than in benign tumors [7,28]. In another study, the author concluded that the DWI-MRI signal was more related to the signal intensity of the fluid rather than to a histopathological component [29]. In our study, except for ovarian cyst, most parts of the primary adnexal lesions showed high signals on *b* = 700 DWI-MRI in both benign and malignant tumors. On the whole, there was significant difference in the DWI-MRI signal between non-malignant and malignant lesions, a finding that is inconsistent with that of a study by Fujii S et al. [30]. The possible explanation is that in their study, teratoma was included, which was different with our studied samples, may result in elevated numbers with high DWI-MRI signal in benign group. In one article, investigators stated that low T2 signal and low signal on DWI-MRI with a *b*-value of 1000 were the best criteria for predicting benignity [9]. However, we do not support this point of view. In our data, we found that few lesions with these characteristics were encountered, which would lead to limited application in a clinical situation.

In theory, as a result of high cell densities and abundant cellular membranes, the ADCs should be generally lower in cancerous tissue than in noncancerous tissue [8]. Even so, the application of ADCs in differentiating malignant from benign ovarian tumors remains a controversy [9,28-32]. The choice of *b*-values, varying pathologies and the number of studied samples all result in a wide divergence of reported ADCs for ovarian cystic and solid diseases. In our results, the highest ADC value was 2.47 ± 0.93 observed in ovarian cyst, while the lowest was 1.39 ± 0.62 in the malignant group. It is worth noting that the wide range of calculated ADCs in the benign group resulted in considerable overlap with those of the endometrioma and malignant tumor, although there was a statistically significant difference between the benign and malignant tumor (*P* = 0.000). This phenomenon reflects the more variable components of benign ovarian tumors. We excluded teratomas from further analysis because these lesions can be easily detected without any diagnostic challenge [9,21,29]. Thomassin NI et al. reported that the ADCs of the cystic component and of the solid component did not differ between malignant and benign adnexal masses [9]. However, in their study, the benign groups with measurable solid component ADCs mainly composed of rich collagen fibril cells obviously restricted the diffusion of water molecules and resulted in low ADCs, which was different with our study. In another study, the authors also declared that ADCs may not provide additional information for differentiating between benign and malignant tumors [29]. However, there were only 10 malignant cases in their research, which considerably limited the broader applicability of their conclusions. In addition, ovarian metastatic tumors were excluded in our patient selection, which is mostly different from the selection used in other reported studies [9,28-32]; therefore, our study sample may more accurately reflect the true features of ovarian masses.

We agree with the view of Takeuchi M et al. that some benign fibrous lesions also have lower ADCs with, ironically, no elevated signal on DWI-MRI [28]. However, the typical character of the low signal on T2WI allows easy differentiation of these benign lesions from malignant tumors. Considering the 22 solid lesions, however, differences in ADCs between benign and malignant types were not observed in this study. Bakir B et al. studied 37 patients with solid or predominantly solid adnexial masses who underwent DWI-MRI evaluation and disclosed that the ADCs of both malignant and benign adnexal lesions also did not show a significant difference [7]. To date, DWI-MRI and ADC evaluations of ovarian solid tumors have not been fully investigated. Because of the relatively small samples, these results are conservative and need to be verified in a large study.

We must acknowledge several limitations in our study. First, some authors have recommended that any lesion displaying a high T1 signal before the DWI sequence should be excluded to limit T1 contamination because the ADC value may increase linearly with decreasing protein concentrations [9,33]. In this study, we did not exclude these lesions so that we could encompass different types of lesions as much as possible. However, we do think this influenced the DWI-MRI signal, which could not show the real restricted diffusion of the lesions. Second, the area of the ROI on which the ADCs were calculated was manually outlined. Lack of standardization in selecting ROI areas also may influence the final ADCs. Third, in our study, we compared our results using 3 T MRI with those of other studies that used 1.5 T MRI. Although Takeuchi M et al. reported that there was no significant difference between the ADC values obtained at 1.5 T and 3.0 T [28], a direct comparison between the two modalities may be still needed to clarify the true difference in ADC s.

## Conclusions

The results of our investigation of 3.0 T MRI revealed that there were no significant differences in MRI signals between benign and malignant lesions. Pure cystic masses are more often observed in benign tumors, while cystic and solid tumors are more common in malignant tumors, as previously reported. DWI-MRI signal and ADCs are useful for discriminating between benign and malignant tumors because the latter show much lower ADC values and high signal on DWI.

## Competing interest

We declare that we have no conflict of interest.

## Author’ contributions

Guarantor of integrity of entire study, GXZ; study concepts/study design or data acquisition or data analysis/interpretation, all authors; manuscript drafting or manuscript revision for important intellectual content, HZ; approval of final version of submitted manuscript, all authors; literature research, HZ; clinical studies, all authors; statistical analysis, HZ, MZ; and manuscript editing, HZ.

## References

[B1] Myers ER BL Havrilesky LJ Cline KE Terplan M Kulasingam SL Gray RN McCrory DC Management of Adnexal Mass Evid Rep Technol Assess (Full Rep) 2006 130 1 145 17854238 PMC4781260

[B2] Rajkotia K Veeramani M Macura KJ Magnetic Resonance Imaging of Adnexal Masses Topics in Magnetic Resonance Imaging 2006 17 379 397 10.1097/RMR.0b013e3180417d8e 17417086

[B3] Griffin N Grant LA Sala E Adnexal Masses: Characterization and Imaging Strategies Seminars in Ultrasound, CT, and MRI 2010 31 330 346 10.1053/j.sult.2010.07.002 20974354

[B4] Hricak H Chen M Coakley FV Complex Adnexal Masses: Detection and Characterization with MR Imaging—Multivariate Analysis1 Radiology 2000 214 39 46 10644099 10.1148/radiology.214.1.r00ja3939

[B5] Sohaib SA Mills TD Sahdev A The role of magnetic resonance imaging and ultrasound in patients with adnexal masses Clinical Radiology 2005 60 340 348 10.1016/j.crad.2004.09.007 15710137

[B6] Chilla B Hauser N Singer G Trippel M Froehlich J Kubik-Huch R Indeterminate adnexal masses at ultrasound: effect of MRI imaging findings on diagnostic thinking and therapeutic decisions European Radiology 2011 21 1301 1310 10.1007/s00330-010-2018-x 21174097

[B7] Bakir B Bakan S Tunaci M Diffusion-weighted imaging of solid or predominantly solid gynaecological adnexial masses: is it useful in the differential diagnosis? British Journal of Radiology 2011 84 600 611 10.1259/bjr/90706205 21081581 PMC3473502

[B8] Levy A Medjhoul A Caramella C Interest of diffusion-weighted echo-planar MR imaging and apparent diffusion coefficient mapping in gynecological malignancies: a review Journal of Magnetic Resonance Imaging 2011 33 1020 1027 10.1002/jmri.22546 21509857

[B9] Thomassin-Naggara I Darai E Cuenod CA Contribution of diffusion-weighted MR imaging for predicting benignity of complex adnexal masses European Radiology 2009 19 1544 1552 10.1007/s00330-009-1299-4 19214523

[B10] Takeuchi M Matsuzaki K Kubo H Nishitani H Magnetic Resonance Manifestations of Endometrial Cysts at 3 T Compared With 1.5 T Journal of Computer Assisted Tomography 2008 32 369 371 10.1097/RCT.0b013e318123e872 18520539

[B11] Kaneda S Fujii S Fukunaga T Myometrial invasion by endometrial carcinoma: evaluation with 3.0 T MR imaging Abdominal Imaging 2011 36 612 618 10.1007/s00261-011-9719-8 21479805

[B12] Komatsu T Konishi I Mandai M Adnexal masses: transvaginal US and gadolinium-enhanced MR imaging assessment of intratumoral structure Radiology 1996 198 109 115 8539360 10.1148/radiology.198.1.8539360

[B13] Grab D Flock F Stöhr I Classification of Asymptomatic Adnexal Masses by Ultrasound, Magnetic Resonance Imaging, and Positron Emission Tomography Gynecologic Oncology 2000 77 454 459 10.1006/gyno.2000.5768 10831359

[B14] Rieber A Nüssle K Stöhr I Preoperative Diagnosis of Ovarian Tumors with MR Imaging American Journal of Roentgenology 2001 177 123 129 11418411 10.2214/ajr.177.1.1770123

[B15] Funt SA Hann LE HL. Detection and characterization of adnexal masses Radiol Clin North Am 2002 40 591 608 10.1016/S0033-8389(01)00009-4 12117195

[B16] Sohaib SAA Sahdev A Trappen PV Jacobs IJ Reznek RH Characterization of Adnexal Mass Lesions on MR Imaging American Journal of Roentgenology 2003 180 1297 1304 12704041 10.2214/ajr.180.5.1801297

[B17] Lee EJ Kim SH Kim YH Lee HJ Is CA-125 an additional help to radiologic findings for differentiation borderline ovarian tumor from stage I carcinoma? Acta Radiologica 2011 52 458 462 10.1258/ar.2011.100318 21498310

[B18] Moyle P Addley HC Sala E Radiological Staging of Ovarian Carcinoma Seminars in ultrasound, CT, and MR 2010 31 388 398 10.1053/j.sult.2010.07.003 20974358

[B19] Shaaban A Rezvani M Ovarian Cancer: Detection and Radiologic Staging Clinical Obstetrics and Gynecology 2009 52 73 93 10.1097/GRF.0b013e3181961625 19179862

[B20] Togashi K Ovarian cancer: the clinical role of US, CT, and MRI European Radiology 2003 13 L87 L104 10.1007/s00330-003-1964-y 15018172

[B21] Bazot M Darai E Nassar-Slaba J Lafont C Thomassin-Naggara I Value of Magnetic Resonance Imaging for the Diagnosis of Ovarian Tumors: A Review Journal of Computer Assisted Tomography 2008 32 712 723 10.1097/RCT.0b013e31815881ef 18830100

[B22] Kinkel K Frei K Balleyguier C Chapron C Diagnosis of endometriosis with imaging: a review European Radiology 2006 16 285 298 10.1007/s00330-005-2882-y 16155722

[B23] Tanaka YO Okada S Yagi T MRI of Endometriotic Cysts in Association With Ovarian Carcinoma American Journal of Roentgenology 2010 194 355 361 10.2214/AJR.09.2985 20093596

[B24] Namimoto T Awai K Nakaura T Yanaga Y Hirai T Yamashita Y Role of diffusion-weighted imaging in the diagnosis of gynecological diseases European Radiology 2009 19 745 760 10.1007/s00330-008-1185-5 18839179

[B25] Thomassin-Naggara I Toussaint I Perrot N Characterization of Complex Adnexal Masses: Value of Adding Perfusion- and Diffusion-weighted MR Imaging to Conventional MR Imaging Radiology 2011 258 793 803 10.1148/radiol.10100751 21193596

[B26] Sala E Rockall A Rangarajan D Kubik-Huch RA The role of dynamic contrast-enhanced and diffusion weighted magnetic resonance imaging in the female pelvis European journal of radiology 2010 76 367 385 10.1016/j.ejrad.2010.01.026 20810230

[B27] Punwani S Diffusion weighted imaging of female pelvic cancers: Concepts and clinical applications European journal of radiology 2011 78 21 29 10.1016/j.ejrad.2010.07.028 20801592

[B28] Takeuchi M Matsuzaki K Nishitani H Diffusion-Weighted Magnetic Resonance Imaging of Ovarian Tumors: Differentiation of Benign and Malignant Solid Components of Ovarian Masses Journal of Computer Assisted Tomography 2010 34 173 176 10.1097/RCT.0b013e3181c2f0a2 20351498

[B29] Katayama M Masui T Kobayashi S Diffusion-Weighted Echo Planar Imaging of Ovarian Tumors: Is It Useful to Measure Apparent Diffusion Coefficients? Journal of Computer Assisted Tomography 2002 26 250 256 10.1097/00004728-200203000-00015 11884782

[B30] Fujii S Kakite S Nishihara K Diagnostic accuracy of diffusion-weighted imaging in differentiating benign from malignant ovarian lesions Journal of Magnetic Resonance Imaging 2008 28 1149 1156 10.1002/jmri.21575 18972356

[B31] Nakayama T Yoshimitsu K Irie H Diffusion-weighted echo-planar MR imaging and ADC mapping in the differential diagnosis of ovarian cystic masses: Usefulness of detecting keratinoid substances in mature cystic teratomas Journal of Magnetic Resonance Imaging 2005 22 271 278 10.1002/jmri.20369 16028258

[B32] Moteki T Ishizaka H Evaluation of cystic ovarian lesions using apparent diffusion coefficient calculated from turboFLASH MR images British Journal of Radiology 1998 71 612 620 9849383 10.1259/bjr.71.846.9849383

[B33] Moteki T Horikoshi H Endo K Relationship between apparent diffusion coefficient and signal intensity in endometrial and other pelvic cysts Magnetic resonance imaging 2002 20 463 470 10.1016/S0730-725X(02)00524-6 12361793

